# A Novel Pathogenic Variant in the RDH5 Gene in a Patient with Fundus Albipunctatus and Severe Macular Atrophy

**DOI:** 10.1155/2022/1183772

**Published:** 2022-04-06

**Authors:** Hyelin You, David Sierpina

**Affiliations:** Department of Ophthalmology, Loma Linda University Eye Institute, Loma Linda, CA, USA

## Abstract

**Purpose:**

To report a novel 11-cis retinol dehydrogenase gene (RDH5) variant discovered in a 57-year-old male with fundus albipunctatus (FA) complicated by severe macular atrophy.

**Methods:**

The patient was evaluated with a complete ophthalmic examination, optical coherence tomography (OCT), color fundus photography, green wavelength fundus autofluorescence, visual field testing, full-field ERG (ffERG), and multifocal ERG (mfERG). Genetic analysis investigating gene variants involved in inherited retinal disorders was performed.

**Results:**

The patient presented with a rapid decline in visual acuity and a history of poor night vision. On fundoscopy, he exhibited a phenotype characteristic of FA accompanied by severe macular atrophy bilaterally. Heterozygous variants in the *RDH5* gene were identified, including a novel missense variant, c.814_815del (p.Leu272Aspfs^*∗*^63), and a known pathogenic nonsense variant, c.160C > T (p.Arg54^*∗*^). Fundus autofluorescence demonstrated bull's eye maculopathy and hyperautofluorescent perifoveal rings bilaterally. OCT showed foveal atrophy of the outer retina and scattered hyper-reflective lesions in the peripheral macula. The ffERG results showed a severely diminished scotopic and photopic response. The mfERG results demonstrated minimal response in the central macula.

**Conclusions:**

Fundus albipunctatus is a rare, congenital form of stationary night blindness caused almost exclusively by the *RDH5* gene. This patient's clinical presentation, diagnostic studies, and genetic testing confirmed the diagnosis of FA. Additionally, he exhibited severe macular atrophy, not typically found in FA. Two *RDH5* gene variants were identified, one of which is the novel variant, c.814_815del (p.Leu272Aspfs^*∗*^63). We suggest that this *RDH5* genotype may be associated with a more progressive phenotype of FA contributing to macular atrophy.

## 1. Introduction

Fundus albipunctatus (FA; OMIM #136880) is a rare, congenital form of stationary night blindness that is characterized by the appearance of numerous small, round, yellow-white lesions throughout the retina, typically found in the retinal periphery and perimacular area while sparing the fovea [1, 2]. Patients with FA classically present with night blindness from early childhood with an otherwise stationary clinical course and normal visual acuity, visual field, and color perception with no progression in rod dysfunction in most patients [3]. For instance, while scotopic full-field electroretinography (ERG) is significantly reduced after a short (30–40 min) period of dark adaptation, the response has been demonstrated to normalize after a prolonged period (2 to 3 hours) of dark adaptation [4–8]. Additionally, the photopic ERG response remains normal in the majority of FA patients.

However, in recent decades, there have been increasing reports of a subset of FA patients who progress to develop macular atrophy and cone dystrophy, particularly in the older population, with a review of the literature by Skorczyk-Werner et al. reporting an incidence of up to 30% [7, 9–11]. Additionally, a recent cohort study by Katagiri et al. which examined 25 Japanese patients with *RDH5*-related FA further demonstrated high frequencies of macular involvement and cone dysfunction in older patients. In this study, 17 of 23 patients (73.9%) examined with full-field ERG demonstrated diminished cone responses in addition to severely decreased rod responses. Macular involvement was noted in 12 patients (48%), 2 of which (8%) had diffuse macular disruption and decreased visual acuity [12]. These studies suggest that FA may have a progressive rather than a stationary course in certain patients.

The pathogenesis of FA at the molecular level can be attributed almost exclusively to a variation in the 11-*cis* retinol dehydrogenase gene (*RDH5*) which produces an enzyme predominantly expressed in the retinal pigment epithelium (RPE) of the eye [6, 13]. Currently, as many as 61 variants of the *RDH5* gene have been identified to date worldwide, with variant types consisting of 46 missense/nonsense variants, 8 small deletions, 4 small insertions/duplications, 2 small indels, and 1 splicing substitution. Of these variants, 49 have been reported in association with a disease phenotype consistent with FA [5–12, 14–27].

Reported cases in the literature demonstrate that different genetic variants can result in a spectrum of phenotypic presentations of FA, as recent studies have suggested a causal relationship between certain *RDH5* genetic variants and cone dystrophy or macular degeneration in addition to nyctalopia [5]. Additionally, certain variants have been associated with an early reduction (as young as ages 6, 19, and 22) in photopic response, suggesting that some variants have a greater negative effect on the cone system [5, 14]. Further characterization of the phenotypic correlation of different *RDH5* variants would be helpful in identifying and counseling the subset of patients that are genetically predisposed to developing early-onset macular atrophy or cone dystrophy.

In this report, we present a patient with FA complicated by severe macular atrophy who was heterozygous for two identified *RDH5* gene variants, one of which is a previously unreported, novel missense variant of presumed pathogenicity, c.814_815del (p.Leu272Aspfs^*∗*^63), as well as a previously described pathogenic nonsense variant, c.160C > T (p.Arg54^*∗*^) also known as c.343C > T in the literature [10]. We also present color fundus photography, green wavelength fundus autofluorescence, and spectral-domain optical coherence tomography (OCT) findings characteristic of FA in this patient, as well as full-field ERG (ffERG) and multifocal ERG (mfERG) results demonstrating macular dysfunction.

## 2. Case Presentation

A 57-year-old Hispanic male was referred for retinal evaluation due to progressive decline of central vision bilaterally, more prominent in the right eye than the left, that occurred over the course of 7 months. He complained of difficulty reading and watching TV as well as bilateral light sensitivity. He denied new distortion in vision, new floaters, flashing lights, head or eye trauma, prior eye surgery, and a history of strabismus or amblyopia. His ophthalmic history was unremarkable aside from poor night vision since childhood. His family history was negative for retinal diseases, and his two children and seven grandchildren denied night vision disturbance. His general medical condition was unremarkable aside from a history of hypertension, well-controlled on lisinopril.

**Figure 1:**
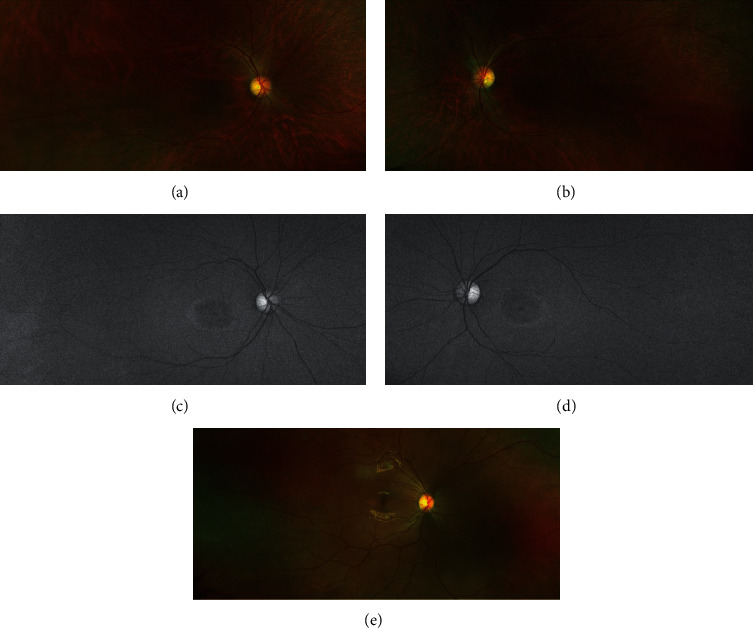
Ultra-wide-field confocal scanning laser ophthalmoscopy (a, b) showing significant macular atrophy with diffuse, fine, yellow flecks bilaterally, as compared to a normal fundus (e); fundus autofluorescence (c, d) demonstrating bull's eye maculopathy and hyperautofluorescent perifoveal rings bilaterally.

**Figure 2:**
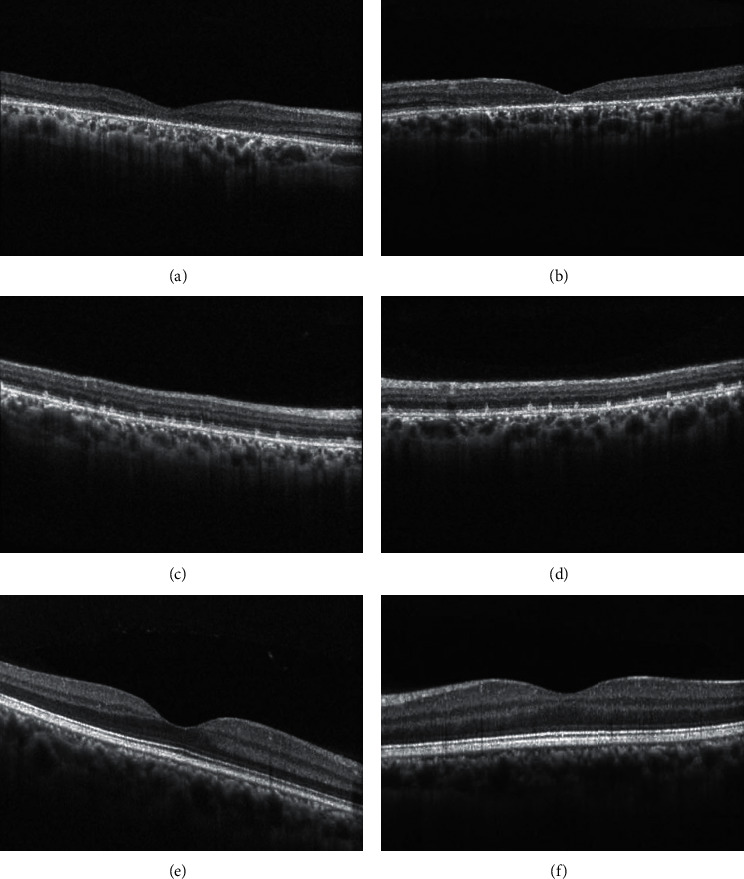
Optical coherence tomography (OCT) shows foveal atrophy of the outer retina extending from the ellipsoid layer to the outer plexiform layer in the right (a) and left (b), and scattered hyper-reflective lesions in the peripheral macula extending from the retinal pigment epithelium to the outer nuclear layer in the right (c) and left (d), as compared to normal OCT of the right (e) and left (f) eyes.

**Figure 3:**
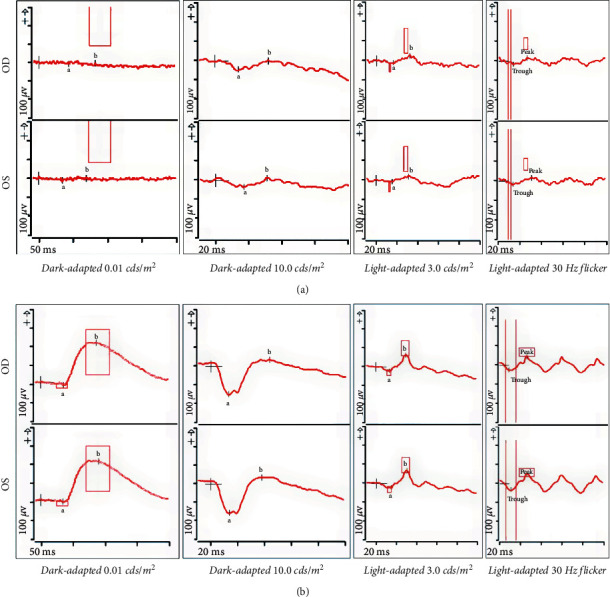
Full-field electroretinography (ERG) demonstrating a severely diminished response to scotopic flash, photopic flash, and 30 Hz flicker stimulation conditions (a), as compared to a normal ERG response in an age-matched control (b), with normative data for amplitude and latencies of age-matched controls represented by red boxes on each panel.

On presentation, visual acuity was measured by counting fingers at 4 feet OD and counting fingers at 3 feet OS. Anterior segment exam, pupillary reflexes, and extraocular movements were normal. Dilated fundus exam as well as ultra-wide-field confocal scanning laser ophthalmoscopy showed significant macular atrophy in both eyes, with 3+ diffuse, fine, yellow flecks (Figures 1(a) and 1(b), Optos California, Marlborough, MA, USA). Green wavelength fundus autofluorescence (Optos California, Marlborough, MA, USA) was remarkable for bull's eye maculopathy and hyperautofluorescent perifoveal rings bilaterally (Figures 1(c) and 1(d)). Optical coherence tomography (OCT, Heidelberg Spectralis, Heidelberg, Germany) showed foveal atrophy of the outer retina extending from the ellipsoid layer to the outer plexiform layer and scattered hyper-reflective lesions in the peripheral macula extending from the retinal pigment epithelium to the outer nuclear layer and corresponding to the flecks observed on fundoscopy (Figure 2). Visual field testing (Humphrey Visual Field Analyzer 3, Carl Zeiss Meditec, Inc., Dublin, CA, USA) was performed but found to be unreliable due to high fixation losses bilaterally. Full-field ERG (ffERG) and multifocal ERG (mfERG) were recorded using the Espion E3 Electroretinography System (Diagnosys LLC, Littleton, Massachusetts, USA) according to ISCEV standards [28]. On ffERG, the responses from both eyes appeared severely diminished to scotopic flash, photopic flash, and 30 Hz flicker stimulation conditions, suggesting severe electroretinal dysfunction of both rod and cone systems in the peripheral and central retina bilaterally, with rod function affected to a greater degree, consistent with rod-cone dystrophy (Figure 3). On mfERG, the trace arrays, ring averages, topographic 3D response density plots, and normal deviation plots appeared severely depressed in both eyes, suggesting severe electroretinal dysfunction of the cone system within the central 30 degrees surrounding the fovea.

Following detailed informed consent, a blood sample was sent to the Invitae Genetic Testing Laboratory (San Francisco, CA, USA) where the inherited retinal disorders panel was performed, consisting of sequence analysis and deletion/duplication testing of 248 genes for variants associated with genetic disorders. Our patient was heterozygous for two variants in the *RDH5* gene: a known pathogenic variant, c.160C > T (p.Arg54^*∗*^), and a novel variant of presumed pathogenicity, c.814_815del (p.Leu272Aspfs^*∗*^63), which previously have not been reported in association with an *RDH5*-related condition. Genetic analysis of the patient's 35-year-old, asymptomatic son revealed heterozygosity for one pathogenic *RDH5* variant, c.160C > T (p.Arg54^*∗*^), demonstrating in-trans segregation of the variant.

The novel *RDH5* gene variant c.814_815del (p.Leu272Aspfs^*∗*^63), while not reported in the literature in individuals with FA, was presumed to be pathogenic by the Invitae Genetic Testing Laboratory, given that this variant disrupts the C-terminus of the RDH5 protein—a region disrupted by other variants reported in association with FA. We confirmed that this variant is present in population databases (rs765714290). Per the Exome Aggregation Consortium database, the reported allele frequency is 8.24e^−6^. This variant is also listed in the ClinVar database under the variation ID 281407, however, without literature evidence for a direct pathogenic association to FA. The reference article listed in ClinVar for this variant describes patients with FA with other *RDH5* variants that disrupt the same C-terminal region of the RDH5 protein as c.814_815del, however, without report of this specific variant [17]. To further verify that this variant has not been previously reported in association with FA, PubMed was searched using the following search terms, without result: “*RDH5* AND c.814_815del,” “*RDH5* AND p.Leu272Aspfs ^*∗*^63,” “c.814_815del,” and “p.Leu272Aspfs ^*∗*^63.” The Human Gene Mutation Database was also searched without a matching result under small deletions and gross deletions in the *RDH5* gene.

## 3. Discussion

Fundus albipunctatus is an autosomal recessive form of stationary night blindness caused primarily by variants of the *RDH5* gene. In most patients, it classically presents as night blindness from early childhood with an otherwise stationary clinical course with normal visual acuity, visual field, and color perception and no further progression to vision loss. However, since the initial description of the stationary clinical course of FA in the early 1920s, there has been an increasing number of reports indicating a more progressive form of the disorder accompanied by macular degeneration or cone dystrophy particularly in the elderly population, as demonstrated in our patient [5, 9, 12, 14, 16]. In these patients, full-field photopic electroretinograms are severely reduced and often demonstrate a bull's eye maculopathy with impaired visual fields and visual acuity [5, 14]. More recent reports suggest that cone dysfunction may affect approximately 30% of all patients with FA [7, 9–11]. Furthermore, one study demonstrated that as many as 17 of 23 patients (73.9%) examined with full-field ERG showed diminished cone responses in addition to severely decreased rod responses [12].

The pathogenesis of FA at the molecular level can be attributed almost exclusively to variants in the *RDH5* gene [6, 13]. The RDH5 protein plays a crucial role in the molecular transduction of vision, as 11-*cis* retinol dehydrogenase catalyzes the final step in the synthesis of 11-*cis* retinaldehyde, the universal chromophore of visual pigments [13]. A disruption in this pathway results in the ocular findings characteristic of FA. Thus, the incorporation of genetic testing in identifying *RDH5* gene variants has facilitated the accurate diagnosis of FA in patients whose phenotypic presentation can be clinically confused with other flecked retina syndromes such as retinitis punctata albescens.

Several genetic variants in the *RDH5* gene have been identified with reports in the literature which suggest that different variants are associated with specific phenotypic presentations of FA. For example, Pras et al. in their case series reported an 18-year-old patient with a missense c.565G > A transition variant who developed cone dysfunction at adolescence, while older middle-aged patients affected by a different variant had not developed functional photoreceptor cell dysfunction at the time of the study [10]. The variables that determine the prognosis of FA are still being investigated, but the available data suggest that one's genotype may play a significant role.

In our patient, two *RDH5* gene variants were identified: c.160C > T (p.Arg54^*∗*^) and c.814_815del (p.Leu272Aspfs ^*∗*^63). The first variant, c.160C > T (p.Arg54^*∗*^), is a nonsense variant resulting in an absent or disrupted protein product, which was previously reported in 2012 by Pras et al. in 10 patients with FA from ages 19 to 55 [10]. The majority of these patients were found to have a stationary disease course without associated cone dystrophy. However, one out of the 10 patients with this specific genetic variant, a 55-year-old male who was homozygous for this variant, was reported to exhibit a gradual development in cone dysfunction. Overall, there was a low incidence of macular atrophy in the majority of patients with this pathogenic variant. Additionally, the presence of this variant in the patient's son resulted in a visually asymptomatic phenotype.

The second gene variant is the novel missense variant c.814_815del (p.Leu272Aspfs^*∗*^63), which results in a frameshift mutation in the *RDH5* gene that disrupts the last 47 amino acids of the RDH5 protein and extends the protein by an additional 15 amino acids. This exact variant has not been previously reported in the literature in individuals with *RDH5*-related conditions such as FA. However, a different pathogenic genetic variant that affects the same region of the RDH5 protein (C-terminus) has been reported in a patient with FA by Liu et al. [17]. They described a nonsense variant, c.832C > T (p.Arg278^*∗*^), in a 6-year-old male patient with FA with early-onset cone dysfunction, suggesting not only the involvement of this variant in the development of FA but that a disruption in this region may also result in a greater negative effect on the cone system compared to other *RDH5* gene variants.

The combined effect of these two pathogenic *RDH5* gene variants in our patient presented as the classic fundus findings of FA as well as macular atrophy that resulted in late-onset, severe, and bilateral vision loss. Given that the known c.160C > T variant does not seem to be associated with macular dysfunction in most patients, this patient's phenotypic presentation may suggest that the novel *RDH5* missense variant, c.814_815del, could be responsible for the macular atrophy and visual dysfunction. This is further supported by the fact that a different known genetic variant affecting the same C-terminal region of the RDH5 protein as the novel variant has also resulted in early-onset of cone dysfunction.

While the specific roles of the various *RDH5* genetic variants is unclear in the pathogenesis of FA, current literature seems to suggest a significant role of genetic variants in determining the prognosis in this patient population. Different variants of the same gene seem to yield a wide array of phenotypic presentations, from isolated stationary night blindness to rapidly progressive total vision loss. Thus, the identification of *RDH5* variants with a higher probability of resultant macular degeneration or cone dystrophy in FA patients is valuable in clinical practice as we provide patients with individualized prognostic information on potential vision loss based on their genotype. We have presented the first reported case of the novel gene variant, c.814_815del (p.Leu272Aspfs^*∗*^63), in an individual with FA, which may exist in a subset of *RDH5* variants associated with macular atrophy or cone dystrophy. To further elucidate the correlation of genotype and phenotype among the various *RDH5* variants, additional data and further work in this area is needed.

## Data Availability

The data are kept in compliance with ethical standards at the Loma Linda University Eye Institute in Loma Linda, California.

## References

[1] Heckenlively J., Traboulsi E. I. (1998). Congenital stationary night blindness. *Genetic Diseases of the Eye*.

[2] Marmor M. F. (1990). Long-term follow-up of the physiologic abnormalities and fundus changes in fundus albipunctatus. *Ophthalmology*.

[3] Gass J. D. M. (1997). *Stereoscopic Atlas of Macular Diseases: Diagnosis and Treatment*.

[4] Dryja T. P. (2000). Molecular genetics of Oguchi disease, fundus albipunctatus, and other forms of stationary night blindness: LVII Edward Jackson Memorial Lecture. *American Journal of Ophthalmology*.

[5] Nakamura M., Skalet J., Miyake Y. (2003). *RDH5* gene mutations and electroretinogram in fundus albipunctatus with or without macular dystrophy: *RDH5* mutations and ERG in fundus albipunctatus. *Documenta Ophthalmologica*.

[6] Yamamoto H., Yakushijin K., Kusuhara S., Escaño M. F., Nagai A., Negi A. (2003). A novel *RDH5* gene mutation in a patient with fundus albipunctatus presenting with macular atrophy and fading white dots. *American Journal of Ophthalmology*.

[7] Sergouniotis P. I., Sohn E. H., Li Z. (2011). Phenotypic variability in *RDH5* retinopathy (fundus albipunctatus). *Ophthalmology*.

[8] Wang N. K., Chuang L. H., Lai C. C. (2012). Multimodal fundus imaging in fundus albipunctatus with *RDH5* mutation: a newly identified compound heterozygous mutation and review of the literature. *Documenta Ophthalmologica*.

[9] Niwa Y., Kondo M., Ueno S., Nakamura M., Terasaki H., Miyake Y. (2005). Cone and rod dysfunction in fundus albipunctatus with *RDH5* mutation: an electrophysiological study. *Investigative Ophthalmology & Visual Science*.

[10] Pras E., Pras E., Reznik-Wolf H. (2012). Fundus albipunctatus: novel mutations and phenotypic description of Israeli patients. *Molecular Vision*.

[11] Skorczyk-Werner A., Pawłowski P., Michalczuk M. (2015). Fundus albipunctatus: review of the literature and report of a novel *RDH5* gene mutation affecting the invariant tyrosine (p.Tyr175Phe). *Journal of Applied Genetics*.

[12] Katagiri S., Hayashi T., Nakamura M. (2020). RDH5-Related fundus albipunctatus in a large Japanese cohort. *Investigative Ophthalmology & Visual Science*.

[13] Simon A., Hellman U., Wernstedt C., Eriksson U. (1995). The retinal pigment epithelial-specific 11-cis retinol dehydrogenase belongs to the family of short chain alcohol dehydrogenases. *Journal of Biological Chemistry*.

[14] Nakamura M., Hotta Y., Tanikawa A., Terasaki H., Miyake Y. (2000). A high association with cone dystrophy in Fundus albipunctatus caused by mutations of the *RDH5* gene. *Investigative Ophthalmology & Visual Science*.

[15] Rüther K., Janssen B. P., Kellner U. (2004). Klinische und molekulargenetische Befunde bei einer Patientin mit Fundus albipunctatus [Clinical and genetic findings in a patient with fundus albipunctatus]. *Ophthalmologe, Der*.

[16] Wada Y., Abe T., Sato H., Tamai M. (2001). A novel Gly35Ser mutation in the *RDH5* gene in a Japanese family with fundus albipunctatus associated with cone dystrophy. *Archives of Ophthalmology*.

[17] Liu X., Liu L., Li H., Xu F., Jiang R., Sui R. (2015). *RDH5* retinopathy (fundus albipunctatus) with preserved rod function. *Retina*.

[18] Lidén M., Romert A., Tryggvason K., Persson B., Eriksson U. (2001). Biochemical defects in 11-cis-retinol dehydrogenase mutants associated with fundus albipunctatus. *Journal of Biological Chemistry*.

[19] Hajali M., Fishman G. A., Dryja T. P., Sweeney M. O., Lindeman M. (2009). Diagnosis in a patient with fundus albipunctatus and atypical fundus changes. *Documenta Ophthalmologica*.

[20] Sekiya K., Nakazawa M., Ohguro H., Usui T., Tanimoto N., Abe H. (2003). Long-term fundus changes due to Fundus albipunctatus associated with mutations in the RDH5 gene. *Archives of Ophthalmology*.

[21] Kuroiwa S., Kikuchi T., Yoshimura N. (2000). A novel compound heterozygous mutation in the RDH5 gene in a patient with fundus albipunctatus. *American Journal of Ophthalmology*.

[22] Schatz P., Preising M., Lorenz B. (2010). Lack of autofluorescence in fundus albipunctatus associated with mutations in RDH5. *Retina*.

[23] Ajmal M., Khan M. I., Neveling K. (2012). Novel mutations in RDH5 cause fundus albipunctatus in two consanguineous Pakistani families. *Molecular Vision*.

[24] Driessen C. A., Janssen B. P., Winkens H. J. (2001). Null mutation in the human 11-cis retinol dehydrogenase gene associated with fundus albipunctatus. *Ophthalmology*.

[25] Beryozkin A., Shevah E., Kimchi A. (2015). Whole Exome sequencing reveals mutations in known retinal disease genes in 33 out of 68 Israeli families with inherited retinopathies. *Scientific Reports*.

[26] Makiyama Y., Ooto S., Hangai M. (2014). Cone abnormalities in fundus albipunctatus associated with RDH5 mutations assessed using adaptive optics scanning laser ophthalmoscopy. *American Journal of Ophthalmology*.

[27] Wang C., Nakanishi N., Ohishi K. (2008). Novel RDH5 mutation in family with mother having fundus albipunctatus and three children with retinitis pigmentosa. *Ophthalmic Genetics*.

[28] McCulloch D. L., Marmor M. F., Brigell M. G. (2015). ISCEV Standard for full-field clinical electroretinography. *Documenta Ophthalmologica*.

